# Influence of Social Media on Sexualized Drug Use and Chemsex Among Chinese Men Who Have Sex With Men: Observational Prospective Cohort Study

**DOI:** 10.2196/17894

**Published:** 2020-07-24

**Authors:** Zixin Wang, Xue Yang, Phoenix K H Mo, Yuan Fang, Tsun Kwan Mary Ip, Joseph T F Lau

**Affiliations:** 1 Centre for Health Behaviours Research JC School of Public Health and Primary Care, Faculty of Medicine The Chinese University of Hong Kong Hong Kong Hong Kong; 2 Shenzhen Research Institute The Chinese University of Hong Kong Shenzhen China; 3 Department of Early Childhood Education Faculty of Education and Human Development The Education University of Hong Kong Hong Kong Hong Kong

**Keywords:** influence of social media, sexualized drug use, chemsex, men who have sex with men, prospective observational cohort study

## Abstract

**Background:**

Sexualized drug use (SDU; the use of any psychoactive substance before or during sexual intercourse) is prevalent among men who have sex with men (MSM) and may aggravate the worsening HIV epidemic in this key population.

**Objective:**

This observational prospective cohort study investigated factors predicting the occurrence of SDU within a 6-month follow-up period among a sample of MSM in Hong Kong. We hypothesized that perceptions related to SDU would mediate the association between the influence of social media/gay social networking apps and SDU during the follow-up period.

**Methods:**

Participants were Chinese-speaking men in Hong Kong, China who had anal intercourse with at least one man in the past year. Among 600 participants who completed the baseline telephone survey, 407 (67.8%) completed another telephone survey 6 months later. Logistic regression models and path analysis were fitted.

**Results:**

At Month 6, 6.9% (28/407) and 4.4% (18/407) of participants reported SDU and chemsex during the follow-up period. After adjustment for significant baseline background variables (use of pre-exposure prophylaxis; history of HIV and other sexually transmitted infections; anal intercourse with nonregular male sex partners, condomless anal intercourse with men, multiple male sex partnerships, and SDU at baseline), three constructs of the Theory of Planned Behavior (TPB) were significantly associated with SDU during the follow-up period: (1) positive attitudes toward SDU (adjusted odds ratio [AOR] 1.19, 95% CI 1.05-1.36), (2) perceived support for SDU from significant others (AOR 1.15, 95% CI 1.01-1.30), and (3) perceived behavioral control of refraining from SDU (AOR 0.76, 95% CI 0.59-0.98). Exposure to information supporting SDU on social media and gay social networking apps was also significantly associated with SDU (AOR 1.11, 95% CI 1.01-1.22). Bootstrapping analyses indicated that social media influence was indirectly associated with SDU through TPB-related perceptions of SDU (β=.04; B=.002, 95% CI 0.001-0.01).

**Conclusions:**

Social media and gay social networking apps may be a major source of influence on MSM’s perceptions and actual behaviors related to SDU.

## Introduction

Psychoactive substance use and HIV are intertwined epidemics among MSM [1]. Sexualized drug use (SDU) refers to the use of any psychoactive substance before or during sexual intercourse [2]. Chemsex is considered a subset of SDU, which is commonly defined as the use of specific drugs (methamphetamine, mephedrone, γ-hydroxybutyrate [GHB/GBL], ketamine and cocaine) before or during sexual intercourse [3-5]. Two published systematic reviews addressed SDU and chemsex among MSM and found these practices prevalent across settings [3,6]. A recent study found that 54% of MSM visiting sexually transmitted infection (STI) clinics in the Netherlands reported SDU [4]. The prevalence of using alcohol or illicit psychoactive substances before or during sexual intercourse was 41% among an online sample of MSM in the United Kingdom [5]. In Hong Kong, China, 12.0% of MSM reported SDU in 2017 [7]. The prevalence of chemsex among MSM ranged from 3% to 35% across countries [3,8-10]. Psychoactive substances such as those used for SDU/chemsex adversely affect users’ capacity to perceive and respond to risks during sexual encounters, and may prompt them to engage in high-risk sexual practices [11]. Studies have consistently shown that SDU and chemsex were associated with a higher risk of condomless anal intercourse (CAI), group sex, and fisting, resulting in a higher risk of HIV and other STIs [3,8,12]. SDU/chemsex among MSM in Hong Kong may aggravate the worsening HIV epidemic in this group (6.54% of MSM were HIV-positive in 2017) [13]. However, there is a lack of effective behavioral interventions targeting SDU/chemsex [14-17].

Factors associated with SDU/chemsex among MSM included those related to sociodemographics (eg, age, ethnicity, income, education level, and employment status), sexual orientation, and utilization of HIV testing and other HIV/STI prevention services [3,5,9]. Studies also showed that MSM with SDU/chemsex experience were more likely to access pre-exposure prophylaxis (PrEP) [10,12]. These factors were considered in this study. Perceptions related to SDU also play important roles, and are important for theory-based interventions to address. Qualitative studies found that a key reason MSM engaged in SDU/chemsex was the belief that psychoactive substances could increase stamina and arousal levels, so that an individual could engage in sex for a sustained period [3]. Other reasons given included the following: overcoming underconfidence, enhancing the emotional connection with sex partners, and coping with stress [3]. Although a qualitative study suggested that MSM are under peer pressure to have SDU and chemsex [18], other social network–related factors (such as fear of loss of partners or friends) deterred MSM from using psychoactive substances before or during sexual intercourse [3]. Only one quantitative study showed that a perceived lower confidence of practicing safe sex consistently was associated with a higher likelihood of chemsex among MSM in the United Kingdom [5]. In this study, we chose the Theory of Planned Behavior (TPB) as the framework to guide our examination of perceptions related to SDU [19]. The TPB has commonly been applied to explain various types of risk behaviors [20] and design various health-related interventions [21]. The TPB specifies that attitudes, subjective norms (ie, whether others approve of certain behaviors), and perceived behavioral control (perceived availability of resources and opportunities that enable a person to perform behaviors) determine health behaviors [19]. This framework is potentially applicable for understanding the factors related to SDU/chemsex among MSM, given findings from previous studies that noted the importance of attitudes, norms, and perceived control on this behavior.

Constructs from the TPB and subsequent behaviors may be shaped by social network–related factors as well as the use of social media and gay social networking apps. In Hong Kong, 96% of residents have access to a smartphone [22]; 85% and 57% use Facebook and Instagram, respectively [23]; and about 70% of local MSM use gay social networking apps to seek sex partners [24]. Social media (eg, Facebook, Twitter, Instagram, Weibo) and location-aware gay social networking apps (eg, Grindr, Jack’d, Blued) have facilitated drug purchasing and seeking SDU and chemsex in one’s geographical location [18,25,26]. Across countries, MSM reported that it was common to encounter information related to SDU/chemsex when browsing these social media and apps [18,25,26]. Many MSM openly solicit chemsex in their online profiles [18,25,26], while others reported being asked to use psychoactive substances or engage in SDU/chemsex by other MSM through these apps [18,25-28]. In addition, posts on social media such as Facebook or Twitter usually portray psychoactive substance use as normative, which may increase intentions to practice SDU or chemsex [29,30]. Despite the potential for social media and apps to increase SDU and chemsex among MSM in Hong Kong, no quantitative studies have investigated the influence of these factors.

In addition to social media and gay social networking apps, peer behaviors may also influence SDU and chemsex practices among MSM. Compared to other HIV key populations, MSM tend to have large and dense social networks [31]. Most Chinese MSM are heavily involved in the MSM community, and have strong ties to each other [32]. As MSM are a highly marginalized community in Hong Kong, individuals within it may perceive a high level of similarity to their MSM peers and may consequently find their peers’ experiences particularly valuable and influential. Prior studies have found that peers within an individual’s social network are the most common source for obtaining psychoactive substances [33,34]. Qualitative studies have suggested that MSM often introduce SDU and chemsex to their peers. Previous studies suggested that a higher number of psychoactive substance users in one’s social network was positively associated with substance use in various populations [35-37], including MSM [36].

The Social Learning Theory posits that people learn by both direct experience and observation [38]. Observation of peers is a major source of influence on people’s health attitudes and behaviors [39]. Given the close relationships and high level of perceived similarity among MSM, observing peer behaviors plays an important role in influencing MSM’s attitudes and behaviors related to SDU/chemsex. Such interactions and observations may occur online and offline. In this study, we hypothesized that being exposed to information supporting SDU on social media/gay apps and observing SDU among peers would influence MSM’s perceptions related to SDU, which would influence the occurrence of SDU.

To address these gaps, this study investigated predictors of SDU within a 6-month follow-up period among a sample of MSM in Hong Kong. Potential predictors measured at baseline included sociodemographics and behavioral characteristics of the participants, perceptions related to SDU based on the TPB, and the influence of social media/gay social networking apps and peers. We further tested the hypothesis that perceptions of SDU would mediate the associations between the influence of social media/gay social networking apps and peers on SDU.

## Methods

### Participants and Data Collection

We conducted an observational prospective cohort study among MSM in Hong Kong, China from April 2018 to July 2019. Inclusion criteria for participants of the cohort study were the following: (1) Chinese-speaking men in Hong Kong, (2) aged 18 years or above, and (3) had anal intercourse with at least one man in the last 12 months. Participants were recruited through multiple sources. A recent mapping exercise was conducted by the government, which identified 12 gay bars and 16 gay saunas in Hong Kong. Upon obtaining the approval of the owners, trained and experienced fieldworkers approached prospective MSM participants in these venues at different time slots during weekdays and weekends. They briefed prospective participants about the study details and gave them an information sheet. The research team also conducted online outreach by posting information about the study periodically as discussion topics on the two gay websites with the highest traffic in Hong Kong. If prospective participants were interested in this study, they could contact the interviewers through private messaging or other means (eg, WhatsApp, telephone, email). Recruitment was supplemented by peer referrals. Participants were guaranteed anonymity during the study, and had the right to end participation in the study at any time. Their refusal or withdrawal from the study would not affect their access to any future services. Verbal consent was obtained instead of written consent to allow participants to maintain anonymity, and the fieldworkers signed a form pledging that the participants had been fully informed about the study. Multiple forms of contact information were obtained to make an appointment to conduct a baseline telephone interview. Trained telephone interviewers confirmed participants’ eligibility and consent to participate in the study, and conducted telephone interviews that took approximately 30 minutes to complete. Another telephone survey was conducted 6 months later. At least 5 follow-up calls were made during different time slots during weekdays and weekends before considering the participant lost to follow-up. Upon completion of both surveys, a HK $50 (US $6.45) supermarket or café coupon was mailed to participants as compensation for their time. Telephone numbers and addresses were cross-checked to avoid repetition. Of the 1131 prospective participants approached through outreach in gay venues (n=211), online recruitment (n=607), and peer referral (n=313), 711 were eligible for participation after screening (venues: n=117; online: n=401; referral: n=193). Of these, 600 provided verbal informed consent and completed the baseline telephone interview (venues: n=85; online: n=345; referral: n=170). The response rate was 84.3%. At Month 6, 407 (67.8%) participants completed the follow-up survey. The Survey and Behavioral Research Ethics Committee of the Chinese University of Hong Kong approved this study (reference number 160051).

### Measures

#### Design of the Questionnaires

A panel consisting of a public health researcher, an epidemiologist, a psychologist, an MSM community member, and a community-based organization worker was formed to develop the questionnaires. The questionnaires were tested by 10 local MSM. Based on their feedback, the questionnaires were then finalized by the panel.

#### Baseline Background Characteristics

Information collected included sociodemographics (age, highest education level attained, current marital status, monthly personal income, and current employment status), sexual orientation; utilization of HIV prevention services and PrEP; history of HIV and other STIs; and sexual behaviors with regular and nonregular male sex partners in the last year. A regular male sex partner (RP) was defined as a stable boyfriend, while a nonregular male sex partner (NRP) was defined as a casual sex partner or a male sex worker.

#### Patterns of SDU/Chemsex

In this study, SDU is defined as the use of any of the following psychoactive substances before or during sexual intercourse: ketamine, methamphetamine, cocaine, cannabis, ecstasy, Dormicum/Halcion/Erimin 5/nonprescription hypnotic drugs, heroin, cough suppressant (not for curing cough), amyl nitrite (popper), GHB/GBL, 5-methoxy-N, N-diisopropyltryptamine (Foxy), and mephedrone. We defined chemsex as the use of ketamine, methamphetamine, cocaine, GHB/GBL, or mephedrone before or during sexual intercourse. Such definitions have been used in a number of studies [3-5]. Participants were asked whether they had used any of the aforementioned psychoactive substances at baseline and Month 6 during different reference periods (baseline: lifetime and past year; Month 6: in the past 6 months).

Participants with SDU experience were asked about behavioral details, including the following: (1) types of psychoactive substance used during sexual intercourse, (2) poly-use of psychoactive substances during sexual intercourse, (3) time since the first episode of SDU, (4) frequency of SDU, (5) CAI during SDU, and (6) sexual risk behaviors during their most recent episode of SDU (ie, the number of participants; the use of alcohol and erectile dysfunction drugs; and the occurrence of CAI or group sex).

#### Influence of Social Media/Gay Social Networking Apps and Peers Measured at Baseline

Participants were asked to report the frequency of their exposure to the following information related to SDU on social media (eg, Facebook, Twitter, Instagram, Weibo) or gay social networking apps (eg, Grindr, Jack’d, Blued) in the last 12 months (eg, “Frequency of exposure to others’ personal experiences supporting psychoactive substance use”) with the following response categories: 1=Never, 2=Seldom, 3=Sometimes, and 4=Always. The Influence of Social Media Scale was formed by summing up individual item scores, with higher scores indicating higher levels of exposure to information supporting SDU from social media and gay social networking apps. The Cronbach α for this scale was .70, and a single factor was identified by exploratory factor analysis, explaining 70.1% of total variance. Additionally, the questionnaire also measured the number of peers in one’s social network who had experience with SDU (response categories: None, 1-2, 3-5, 6-10, and >10).

#### Perceptions Related to SDU Based on the TPB

Five scales derived from the TPB were constructed [40]. Positive attitudes toward SDU were measured by four items (eg, “It is easier for you to find sex partners when using psychoactive substances before/during sexual intercourse”). The Positive Attitude Scale was formed by summing up individual item scores (from 1=Strongly disagree to 5=Strongly agree). Higher scores on the scale indicated more positive attitudes toward SDU. The Cronbach α of the Positive Attitude Scale was .82; one factor was identified by exploratory factor analysis, explaining 65.1% of total variance.

Four items (with response options ranging from 1=Strongly disagree to 5=Strongly agree) were used to measure negative attitudes toward SDU (eg, “Using psychoactive substances before/during sexual intercourse would increase your risk of having CAI”). The Negative Attitude Scale was formed by summing up individual item scores. Higher scores on the scale indicated more negative attitudes toward SDU. The Cronbach α of the Negative Attitude Scale was .62; one factor was identified by exploratory factor analysis, explaining 63.0% of total variance.

Four items were used to measure participants’ perceived support from their significant others (referring to regular and nonregular male sex partners and MSM friends) for SDU. Items were measured on a 5-point Likert Scale from 1=Strongly disagree to 5=Strongly agree. The Perceived Subjective Norms for SDU among MSM Scale was constructed by summing up individual item scores. Higher scores indicated perceived subjective norms that were more supportive of SDU. The Cronbach α of the Perceived Subjective Norm Scale was .90; one factor was identified by exploratory factor analysis, explaining 76.8% of total variance.

Perceived behavioral control in refusing SDU and perceived behavioral control in refraining from SDU were measured by two items each. The Perceived Behavioral Control of Refusing SDU Scale and the Perceived Behavioral Control of Refraining from SDU Scale were formed by summing up individual item scores (from 1=Strongly disagree to 5=Strongly agree). Higher scores of the Perceived Behavioral Control of Refusing SDU Scale indicated lower levels of control of refusing SDU, while higher scores in the Perceived Behavioral Control of Refraining from SDU indicated higher levels of control of refraining from SDU. The Cronbach α of the Perceived Behavioral Control of Refusing SDU Scale and the Perceived Behavioral Control of Refraining from SDU Scale were .93 and .95, respectively; single factors were identified by exploratory factor analysis, explaining 93.5% and 95.6% of total variance, respectively.

### Data Analyses

Baseline background characteristics of participants who were followed up at Month 6 and those who were lost to follow-up were compared using chi-square tests (for categorical variables) or independent sample *t* tests (for continuous variables). The subsequent analysis was performed among those who had completed both surveys. Using SDU during the follow-up period as the dependent variable, and background characteristics measured at baseline as independent variables, univariate odds ratios (OR) predicting the dependent variable were obtained using logistic regression models. After adjusting for variables with *P*<.05 in the univariate analysis, associations between independent variables of interest (ie, perceptions, the influence of social media and gay social networking apps and peers) and the dependent variable were then assessed by adjusted OR (AOR). Each AOR was obtained by fitting a single logistic regression model, which involved one of the independent variables of interest and the significant background variables.

Path analysis was conducted to test the mediation model. The means for social media influence and peer influence were used as independent variables, while SDU during the follow-up period was used as the dependent variable. The means of the Positive Attitude Scale, the Negative Attitude Scale, the Perceived Subjective Norm Scale, the Perceived Behavioral Control for Refusing SDU Scale, and the Perceived Behavioral Control for Refraining from SDU Scale were used as indicators to represent the latent variable of perceptions related to SDU based on the TPB; confirmatory factor analysis was then conducted to test the goodness of fit of this construct. All significant background variables were controlled for in the model. Goodness of fit was tested by using the chi-square test, the Comparative Fit Index (CFI), the Non-Normed Fit Index (NNFI), and the root mean square error of approximation (RMSEA). A CFI and NNFI greater than .90 and RMSEA lower than .08 indicated acceptable goodness of fit [41]. Standardized path coefficients (*β*) and unstandardized path coefficients (B) were reported. We tested the mediation analyses using bootstrapping. The 95% CI of the indirect effects were obtained from 5000 bootstrap samples. A statistically significant mediation effect was observed when the CI did not include zero. The level of statistical significance was .05. SPSS (Version 18.0, IBM Corp) and AMOS were used.

## Results

### Baseline Background Characteristics

Most of the 600 participants were 18 to 30 years old (n=342, 57.0%), currently single (n=504, 84.0%), employed full-time (n=498, 83.0%), with a monthly personal income of HK $20,000 (US $2580) or higher (n=336, 56.0%), and had attained at least a college education (n=505, 84.2%). Over half had received HIV antibody testing (n=430, 71.7%) and utilized other HIV prevention services (n=335, 55.8%) in the last year; 3.8% (n=23) were using PrEP at the time of the baseline survey. Among the participants, 3.3% (n=20) self-reported as living with HIV and 20.5% (n=123) had a history of other STIs. In the last year, 85.0% (n=510) and 60.5% (n=363) had had anal intercourse with RP and NRP, respectively. Furthermore, 40.2% (n=241) and 69.0% (n=414) reported CAI with men and multiple sex partnerships, respectively. The lifetime prevalence of SDU and chemsex were 19.3% (n=116) and 8.7% (n=52), respectively. Means and standard deviation of items/scales related to the influence of social media/gay apps, the influence of peers, and perceptions related to SDU based on the TPB were described in Table 1. No significant difference was found in baseline characteristics between those with follow-up data available (n=407) and those who were lost to follow-up at Month 6 (n=193), with the exception of self-reported HIV status (*P*=.04), the frequency of exposure to commentary/discussion about psychoactive substance use on social media/gay apps (*P*=.001), and the score on the Perceived Behavioral Control of Refraining from SDU Scale (*P*=.04).

**Table 1:** Baseline characteristics of the participants.

Characteristics			All participants (n=600)	Follow-up at Month 6 (n=407)	Loss to follow-up (n=193)	*P* value
**Sociodemographics**						
	**Age group (years), n (%)**					**.29**
		18-24	124 (20.7)	80 (19.7)	44 (22.8)	
		25-30	218 (36.3)	146 (35.9)	72 (37.3)	
		31-40	187 (31.2)	126 (31.0)	61 (31.6)	
		>40	71 (11.8)	55 (13.5)	16 (8.3)	
	**Highest educational level attained, n (%)**					**.73**
		Senior high school or below	95 (15.8)	63 (15.5)	32 (16.6)	
		College or above	505 (84.2)	344 (84.5)	161 (83.4)	
	**Current marital status, n (%)**					**.38**
		Currently single	504 (84.0)	344 (84.5)	160 (82.9)	
		Married/cohabiting with a man	93 (15.5)	60 (14.7)	33 (17.1)	
		Married/cohabiting with a woman	3 (0.5)	3 (0.7)	0 (0.0)	
	**Monthly personal income (HK $), n (%)**					**>.99**
		<10,000 (<US $1290)	84 (14.0)	57 (14.0)	27 (14.0)	
		10,000-19,999 (US $1290-2580)	174 (29.0)	120 (29.5)	54 (28.0)	
		20,000-39,999 (US $2580-3870)	220 (36.7)	148 (36.4)	72 (37.3)	
		>40,000 (>US $5161)	116 (19.3)	78 (19.2)	38 (19.7)	
		Refuse to disclose	6 (1.0)	4 (1.0)	2 (1.0)	
	**Current employment status, n (%)**					**.61**
		Full-time	498 (83.0)	340 (83.5)	158 (81.9)	
		Part-time/unemployed/retired/students	102 (17.0)	67 (16.5)	35 (18.1)	
	**Sexual orientation, n (%)**					**.35**
		Homosexual	546 (91.0)	375 (92.1)	171 (88.6)	
		Bisexual	52 (8.7)	31 (7.6)	21 (10.9)	
		Heterosexual	2 (0.3)	1 (0.3)	1 (0.5)	
**Service utilization, n (%)**						
	**HIV testing in the last 12 months**					**.15**
		No	170 (28.3)	108 (26.5)	62 (32.1)	
		Yes	430 (71.7)	299 (73.5)	131 (67.9)	
	**Other HIV prevention services in the last 12 months (eg, condom distribution, peer education, pamphlet and lectures)**					**.46**
		No	265 (44.2)	184 (45.2)	81 (42.0)	
		Yes	335 (55.8)	223 (54.8)	112 (58.0)	
	**Currently on PrEP (pre-exposure prophylaxis)**					**.78**
		No	577 (96.2)	392 (96.3)	185 (95.9)	
		Yes	23 (3.8)	15 (3.7)	8 (4.1)	
**History of HIV/sexually transmitted infections, n (%)**						
	**Self-reported HIV status**					**.04**
		Never tested for HIV	26 (4.3)	11 (2.7)	15 (7.8)	
		Negative	549 (91.5)	380 (93.4)	169 (87.6)	
		Positive	20 (3.3)	13 (3.2)	7 (3.6)	
		Refuse to disclose	5 (0.8)	3 (0.7)	2 (1.0)	
	**History of other sexually transmitted infections**					**.76**
		No	477 (79.5)	325 (79.9)	152 (78.8)	
		Yes	123 (20.5)	82 (20.1)	41 (21.2)	
**Sexual behaviors in the last 12 months, n (%)**						
	**Has had anal intercourse with regular male sex partners**					**.14**
		No	90 (15.0)	55 (13.5)	35 (18.1)	
		Yes	510 (85.0)	352 (86.5)	158 (81.9)	
	**Has had anal intercourse with nonregular male sex partners**					**.69**
		No	237 (39.5)	163 (40.0)	74 (38.3)	
		Yes	363 (60.5)	244 (60.0)	119 (61.7)	
	**Condomless anal intercourse with men**					**.33**
		No	359 (59.8)	249 (61.2)	110 (57.0)	
		Yes	241 (40.2)	158 (38.8)	83 (43.0)	
	**Multiple male sex partnerships**					**.88**
		No	186 (31.0)	127 (31.2)	59 (30.6)	
		Yes	414 (69.0)	280 (68.8)	134 (69.4)	
**Experience of sexualized drug use (SDU)^a^, n (%)**						
	**SDU in lifetime**					**.61**
		No	484 (80.7)	326 (80.1)	158 (81.9)	
		Yes	116 (19.3)	81 (19.9)	35 (18.1)	
	**SDU in the past year**					**.68**
		No	512 (85.3)	349 (85.7)	163 (84.5)	
		Yes	88 (14.7)	58 (14.3)	30 (15.5)	
**Influence of social media related to SDU**						
	**Frequency of exposure to information supporting SDU on social media/gay social networking apps in the past year, % Sometimes/Always**					
		Sharing of personal experiences to support MSM using psychoactive substances	253 (42.2)	168 (41.3)	85 (44.0)	.52
		Sharing of personal experiences against MSM using psychoactive substances (reverse coded)	180 (30.0)	131 (32.2)	49 (25.4)	.09
		Receiving personal invitations to use psychoactive substances from MSM friends	93 (15.5)	58 (14.3)	35 (18.1)	.22
		Receiving personal invitations to have SDU/chemsex from MSM friends	90 (15.0)	55 (13.5)	35 (18.1)	.14
		Receiving personal invitations to use psychoactive substances from strangers	212 (35.3)	145 (35.6)	67 (34.7)	.83
		Receiving personal invitations to have SDU/chemsex from strangers	218 (36.3)	150 (36.9)	68 (35.2)	.70
		Commentary/discussion about psychoactive substance use	168 (28.0)	97 (23.8)	71 (36.8)	.001
	The Influence of Social Media Scale, mean (SD)		7.7 (4.4)	7.5 (4.3)	8.1 (4.4)	.13
**Influence of peers related to** **SDU**						
	Regular male sex partners had experience of SDU/chemsex, % Yes		48 (8.0)	39 (9.6)	9 (4.7)	.04
	Nonregular male sex partners had experience of SDU/chemsex, % Yes		109 (18.2)	72 (17.7)	37 (19.2)	.66
	Close friends had experience of SDU/chemsex, % Yes		129 (21.5)	94 (23.1)	35 (18.1)	.17
	Other friends had experience of SDU/chemsex, % Yes		229 (38.2)	151 (37.1)	78 (40.4)	.44
	**Number of peers in one’s social network who had ever engaged in SDU/chemsex, n (%)**					**.62**
		0	178 (29.7)	121 (29.7)	57 (29.5)	
		1-2	148 (24.7)	104 (25.6)	44 (22.8)	
		3-5	143 (23.8)	92 (22.6)	51 (26.4)	
		6-10	48 (8.0)	36 (8.8)	16 (8.2)	
		>10	83 (13.8)	54 (13.3)	29 (15.0)	
**Perceptions related to SDU based on the Theory of Planned Behavior**						
	**Positive attitudes toward SDU, % Agree/Strongly agree**					
		SDU allows you temporary escape from reality	106 (17.7)	70 (17.2)	36 (18.7)	.66
		SDU increases your sexual pleasure	139 (23.2)	99 (24.3)	40 (20.7)	.33
		It is easier to find sex partners during SDU	74 (12.3)	47 (11.5)	27 (14.0)	.40
		SDU would heighten euphoria	131 (21.8)	93 (22.9)	38 (19.7)	.38
	Positive Attitude Scale, mean (SD)		8.0 (4.2)	8.8 (4.1)	8.8 (4.3)	.93
	**Negative attitudes toward SDU, % Agree/Strongly agree**					
		SDU would harm your cognitive function	558 (94.0)	379 (93.1)	185 (95.9)	.19
		SDU would have negative impact on your relationship with sex partners	384 (64.0)	265 (65.1)	119 (61.7)	.41
		SDU would increase your risk of having condomless anal intercourse	381 (63.5)	258 (63.4)	123 (63.7)	.94
		SDU would increase your risk of HIV infection	511 (85.2)	341 (83.8)	170 (88.1)	.17
	Negative Attitude Scale, mean (SD)		16.8 (2.9)	16.7 (2.9)	16.8 (2.8)	.72
	**Perceived subjective norms related to SDU, % Agree/Strongly agree**					
		Your male sex partners support you using psychoactive substances	29 (4.8)	19 (4.7)	10 (5.2)	.78
		Your other MSM friends support you using psychoactive substances	17 (2.8)	12 (2.9)	5 (2.6)	.81
		Your male sex partners support you having SDU	21 (3.5)	14 (3.4)	7 (3.6)	.91
		Your other MSM friends support you having SDU	11 (1.8)	8 (2.0)	3 (1.6)	.73
	Perceived Subjective Norms Scale, mean (SD)		5.9 (3.1)	5.8 (3.1)	6.0 (3.0)	.40
	**Perceived behavioral control of refusing SDU, % Agree/Strongly agree**					
		If your sex partner asks you to use psychoactive substances, it is difficult for you to refuse	60 (10.0)	36 (8.8)	24 (12.4)	.17
		If your sex partner asks you to have SDU, it is difficult for you to refuse	55 (9.2)	31 (7.6)	24 (12.4)	.06
	Perceived Behavioral Control of Refusing SDU Scale, mean (SD)		3.5 (2.2)	3.4 (2.1)	3.7 (2.3)	.10
	**Perceived behavioral control of refraining from SDU, % Agree/Strongly agree**					
		You can exercise self-control to stop using psychoactive substance	541 (90.2)	371 (91.2)	170 (88.1)	.24
		You can exercise self-control to stop having SDU	550 (91.7)	378 (92.9)	172 (89.1)	.12
	Perceived Behavioral Control of Refraining from SDU Scale, mean (SD)		9.2 (1.7)	9.3 (1.5)	9.0 (2.0)	.04

^a^SDU: sexualized drug use. Sexualized drug use is defined as the use of any of the following psychoactive substances before/during anal intercourse: ketamine, methamphetamine, cocaine, cannabis, ecstasy, Dormicum/Halcion/Erimin 5/nonprescription hypnotic drugs, heroin, cough suppressant (not for curing cough), amyl nitrite (popper), GHB/GBL (γ-hydroxybutyrate), 5-methoxy-N, N-diisopropyltryptamine (Foxy), and mephedrone.

### Patterns of SDU and Chemsex at Baseline and Month 6

At baseline, 14.7% (88/600) and 6.7% (40/600) of the participants reported SDU and chemsex in the past year, respectively. During the 6-month follow-up period, their prevalence was 6.9% (28/407) and 4.4% (18/407), respectively. Among 58 participants with experience of SDU who completed the Month 6 follow-up survey, 36% (n=21) reported SDU during the follow-up period. Among 26 MSM who had chemsex at baseline and completed the Month 6 follow-up survey, 14 (54%) reported chemsex during the follow-up period.

Patterns of SDU one year prior to the baseline and during the 6-month follow-up period were similar. Amyl nitrite, methamphetamine, and GHB/GBL were the most commonly used psychoactive substances during sexual intercourse. About half of MSM who reported SDU reported poly-use of psychoactive substances and CAI. SDU commonly involved more than two people, the use of erectile dysfunction drugs, and group sex (Table 2).

**Table 2:** Patterns of sexualized drug use in different reference periods.

Variables			One year prior to baseline survey (among MSM^a^ who reported sexualized drug use in the past year at baseline, n=88), n (%)	During the 6-month follow-up period (among MSM who reported sexualized drug use during the follow-up, n=28), n (%)
**Types of psychoactive substance used during chemsex**				
	Ketamine		2 (2.3)	2 (7.1)
	Methamphetamine		32 (36.4)	11 (39.3)
	Cocaine		0 (0.0)	0 (0.0)
	Cannabis		7 (8.0)	1 (3.6)
	Ecstasy		2 (2.3)	2 (7.1)
	Dormicum/Halcion/Erimin 5/Hypnotic drugs (nonprescription)		0 (0.0)	1 (3.6)
	Heroin		0 (0.0)	0 (0.0)
	Cough suppressant (not for curing cough)		1 (1.1)	2 (7.1)
	Amyl nitrite		69 (78.4)	20 (71.4)
	γ-hydroxybutyrate (GHB/GBL)		29 (33.0)	10 (35.7)
	5-methoxy-N, N-diisopropyltryptamine		5 (5.7)	1 (3.6)
	Mephedrone		0 (0.0)	1 (3.6)
**Poly-use of psychoactive substances**				
	No		57 (64.8)	14 (50.0)
	Yes		31 (35.2)	14 (50.0)
**Frequency of** **sexualized drug use**				
	<1 episode/month		0 (0.0)	13 (46.4)
	1 episode/month		35 (39.8)	11 (39.3)
	1-2 episodes/month		24 (27.3)	2 (7.1)
	≥3 episodes/month		29 (33.0)	2 (7.1)
**Condomless anal intercourse during** **sexualized drug use**				
	No		41 (46.6)	10 (35.7)
	Yes		47 (53.4)	18 (64.3)
**Characteristics of most recent episode of** **sexualized drug use**				
	**Number of participants**			
		2	67 (76.1)	19 (67.9)
		≥3	21 (23.9)	9 (32.1)
	**Alcohol consumption**			
		No	77 (87.5)	28 (100.0)
		Yes	11 (12.5)	0 (0.0)
	**Use of erectile dysfunction drugs**			
		No	62 (70.5)	16 (57.9)
		Yes	26 (29.5)	12 (42.1)
	**Group sex**			
		No	18 (20.5)	11 (39.3)
		Yes	70 (79.5)	17 (60.7)
	**Condomless anal intercourse**			
		No	45 (51.1)	15 (53.6)
		Yes	43 (48.9)	13 (46.4)

^a^MSM: men who have sex with men.

### Baseline Factors Predicting SDU During the Follow-Up Period

Baseline background variables that were significantly associated with SDU during the follow-up period included the following: (1) currently on PrEP, (2) self-reported living with HIV, (3) history of other STI, (4) any anal intercourse with NRP, (5) CAI with any male sexual partners, (6) multiple male sex partnerships, and (7) SDU in the past year (Table 3).

After adjusting for significant variables, three constructs of the TPB measured at baseline were significantly associated with SDU during the follow-up period. These constructs included: (1) positive attitudes toward SDU (AOR 1.19, 95% CI 1.05-1.36), (2) perceived support for SDU from significant others (AOR 1.15, 95% CI 1.01-1.30), and (3) perceived behavioral control of refraining from SDU (AOR 0.76, 95% CI 0.59-0.98). A higher level of exposure to information supporting SDU on social media/gay apps as measured at baseline was associated with a higher likelihood of SDU during the follow-up period (AOR 1.11, 95% CI 1.01-1.22). The association between the number of peers in one’s social network who had ever engaged in SDU and the dependent variable was of marginal statistical significance (AOR 1.43, 95% CI 0.95-2.16, *P*=.06; Table 4.

**Table 3:** Baseline background variables associated with sexualized drug use during the follow-up period for MSM in Hong Kong (among those being followed up at Month 6, n=407).

Variables			Participants, n (%)	OR^a^ (95% CI)
**Sociodemographics measured at baseline**				
	**Age group (years)**			
		18-24	4 (5.0)	1.0
		25-30	10 (6.8)	1.40 (0.42-4.61)
		31-40	11 (8.7)	1.82 (0.56-5.92)
		>40	3 (5.5)	1.10 (0.24-5.10)
	**Highest educational level attained**			
		Senior high school or below	7 (11.1)	1.0
		College or above	21 (6.1)	0.52 (0.21-1.28)
	**Current marital status**			
		Currently single	24 (7.0)	1.0
		Married/cohabiting with a man	3 (5.0)	0.70 (0.21-2.41)
		Married/cohabiting with a woman	1 (33.3)	6.67 (0.58-76.18)
	**Monthly personal income (HK $)**			
		<10,000 (<US $1290)	5 (8.8)	1.0
		10,000-19,999 (US $1290-2580)	6 (5.0)	0.55 (0.16-1.88)
		20,000-39,999 (US $2580-3870)	12 (8.1)	0.92 (0.31-2.73)
		>40,000 (>US $5161)	5 (6.4)	0.71 (0.20-2.59)
		Refuse to disclose	0 (0.0)	N/A^b^
	**Current employment status**			
		Full-time	21 (6.2)	1.0
		Part-time/unemployed/retired/student	7 (10.4)	1.77 (0.72-4.35)
	**Sexual orientation**			
		Homosexual	27 (7.2)	1.0
		Bisexual	1 (3.2)	0.43 (0.05-3.27)
		Heterosexual	0 (0.0)	N/A
**Service utilization measured at baseline**				
	**HIV testing in the last 12 months**			
		No	4 (3.7)	1.0
		Yes	24 (8.0)	2.27 (0.77-6.70)
	**Other HIV prevention services in the last 12 months (eg, condom distribution, peer education, pamphlets, lectures)**			
		No	12 (6.5)	1.0
		Yes	16 (7.2)	1.11 (0.51-2.41)
	**Currently on PrEP (pre-exposure prophylaxis)**			
		No	23 (5.9)	1.0
		Yes	5 (33.3)	8.02 (2.53-25.42)^c^
**History of HIV/sexual transmitted infections**				
	**Self-reported HIV status**			
		Negative/never tested for HIV/refuse to disclose	24 (6.1)	1.0
		Positive	4 (30.8)	6.85 (1.97-23.87)^d^
	**History of other sexually transmitted infections**			
		No	16 (4.9)	1.0
		Yes	12 (14.6)	3.31 (1.50-7.31)^d^
**Sexual behaviors in the last 12 months as measured at baseline**				
	**Anal intercourse with regular male sex partners**			
		No	2 (3.6)	1.0
		Yes	26 (7.4)	2.11 (.49-9.17)
	**Anal intercourse with nonregular male sex partners**			
		No	1 (.6)	1.0
		Yes	27 (11.1)	20.16 (2.71-149.88)^d^
	**Condomless anal intercourse with men**			
		No	8 (3.2)	1.0
		Yes	20 (12.7)	4.37 (1.87-10.18)^d^
	**Multiple male sex partnerships**			
		No	0 (0)	N/A
		Yes	28 (10.0)	N/A^e^
**Experience of sexualized drug use as measured at baseline**				
	**Sexualized drug use in the past year**			
		No	7 (2.0)	1.0
		Yes	21 (36.2)	27.73 (11.05-69.60)^c^

^a^OR: univariate odds ratio.

^b^N/A: not applicable.

^c^*P*<.001.

^d^*P*<.01.

^e^*P*<.05.

**Table 4:** Factors associated with sexualized drug use during the follow-up period (among those being followed up at Month 6, n=407)^a^.

Factors		OR^b^ (95% CI)	AOR^c^ (95% CI)
**Influence of social media/gay apps related to** **sexualized drug use**			
	Influence of Social Media Scale	1.23 (1.13, 1.34)^d^	1.11 (1.01, 1.22)^e^
**Influence of peers related to** **sexualized drug use**			
	Number of peers in one’s social network who had ever engaged in SDU/chemsex	2.29 (1.67, 3.14)^d^	1.43 (0.95, 2.16)^f^
**Perceptions related to** **sexualized drug use** **based on the Theory of Planned Behavior**			
	Positive Attitude Scale	1.33 (1.19, 1.47)^d^	1.19 (1.05, 1.36)^g^
	Negative Attitude Scale	0.86 (0.76, 0.97)^e^	0.99 (0.84, 1.16)
	Perceived Subjective Norm Scale	1.29 (1.18, 1.42)^d^	1.15 (1.01, 1.30)^e^
	Perceived Behavioral Control of Refusing Sexualized Drug Use Scale	1.33 (1.15, 1.55)^d^	1.08 (0.89, 1.33)
	Perceived Behavioral Control of Refraining from Sexualized Drug Use Scale	0.76 (0.64, 0.91)^g^	0.76 (0.59, 0.98)^e^

^a^6.9% of participants reported sexualized drug use during the follow-up period.

^b^OR: univariate odds ratio. The OR represents the increase in the odds of sexualized drug use caused by a one-unit increase in the item/scale score.

^c^AOR: adjusted odds ratio. The AOR is adjusted for the significant background variables listed in Table 3 and multiple sex partnerships in the past year.

^d^*P*<.001.

^e^*P*<.05.

^f^.05<*P*<.10.

^g^*P*<.01.

### Testing the Mediation Effects of TPB Constructs in the Association Between Social Media/Peers Influences and SDU

#### Model Testing

**Figure 1:**
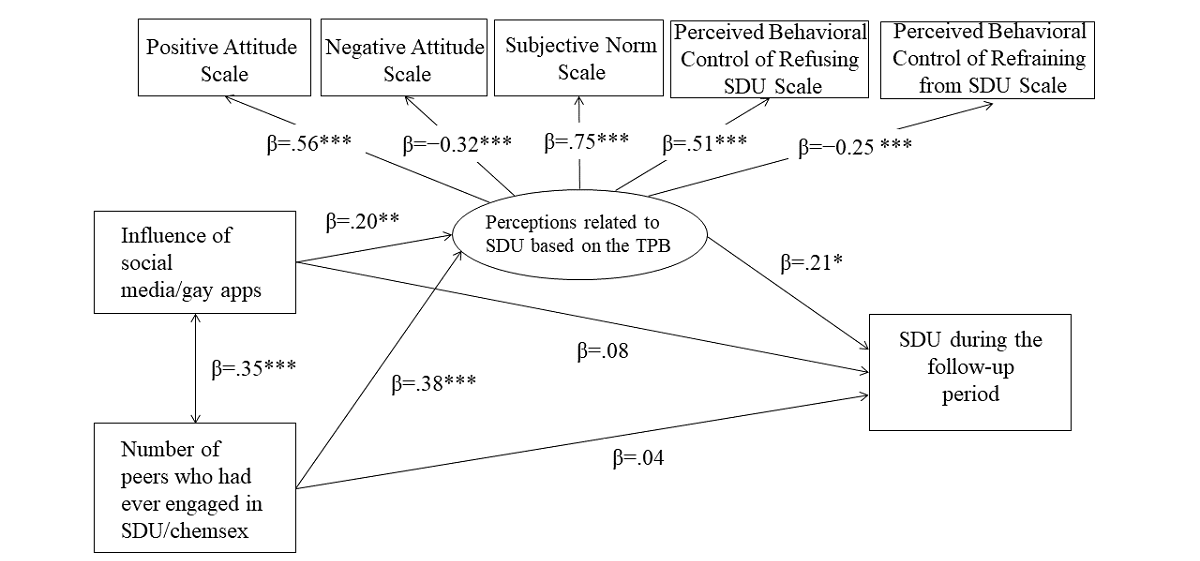
The proposed mediation models with standardized path coefficients. *P<.05; **P<.01; ***P<.001. SDU: sexualized drug use; TPB: Theory of Planned Behavior.

The results of the confirmatory factor analysis found that perceptions related to SDU based on the TPB did not show adequate model fit to the data (*χ*^2^_5_=19.01, *P*<.01; CFI=.94; NNFI=.89; RMSEA=.08). Modification indices suggested adding a covariance between the Perceived Behavioral Control of Refusing SDU Scale’s error and the Perceived Behavioral Control of Refraining from SDU Scale’s error (B=–0.58, *P*<.001). Since the two scales are related constructs [19], we added the covariance accordingly. The modified model showed excellent fit (*χ*^2^_4_=3.81, *P*=.43; CFI=.99; NNFI=.99; RMSEA=.01). The chi-square test showed that the model fit change was significant (*χ*^2^ change=15.20, degrees of freedom change=1, *P*<.05). All factor loadings were significant at *P*<.001, with absolute values of standardized coefficients greater than .25. The mediation model fit the data well (*χ*^2^_147_=188.71, *P*<.05; CFI=.92; NNFI=.91; RMSEA=.06; Figure 1).

#### Path Coefficients

The influence of social media/gay apps was positively associated with perceptions related to SDU based on the TPB (*β*=.20, B=.11, *P*=.001), while it was not significantly associated with SDU (*β*=.08, B=.004, *P*=.11). Peer influence was positively associated with perceptions related to SDU based on the TPB (*β*=.38, B=.68, *P<*.001), while it was not significantly associated with SDU (*β*=.04, B=.01, *P*=.50). Perceptions related to SDU based on the TPB was positively associated with SDU (*β*=.21, B=.02, *P*=.02).

#### Mediation Effects

Bootstrapping analyses indicated that social media influence had a significant indirect effect on SDU through perceptions of SDU in the hypothesized direction (*β*=.04, B=.002, 95% CI 0.001-0.01). However, peer influence was not significantly associated with SDU through perceptions of SDU (*β*=.08, B=.01, 95% CI –0.002 to 0.04).

## Discussion

We found that 6.9% and 4.4% of MSM in Hong Kong practiced SDU and chemsex at the end of the 6-month follow-up period. Such prevalence was lower than that reported among MSM in Western Europe. At Month 6, about half of participants having SDU or chemsex at baseline refrained from these behaviors, while very few initiated these behaviors. Studies with longer follow-up periods are needed in the future to understand trends in SDU and chemsex among MSM in Hong Kong. Such studies are useful to understand the effectiveness of SDU/chemsex interventions conducted by local community-based organizations.

Similar to studies conducted in Western countries, amyl nitrite, methamphetamine, and GHB/GBL were the most commonly used psychoactive substances during SDU at both baseline and Month 6 [7]. The prevalence of risky practices during SDU (ie, poly-use of psychoactive substance, CAI, and group sex) was high at both time points. Effective risk reduction strategies should be explored to address these risky practices.

According to social marketing approaches, careful segmentation improves the effectiveness of health promotion programs [42]. First, heath care workers and interventions should tailor SDU and chemsex reduction measures to MSM living with HIV. Similar to findings conducted in mainland China [43] and other countries [3], MSM living with HIV were more likely to practice SDU compared to HIV-negative or status unknown MSM. They have a high risk of HIV transmission through SDU. For MSM living with HIV, SDU may be a potential maladaptive coping method for stressful situations caused by perceived stigma and experiences of violence and prejudice [44]. Reducing substance use during sex should be an essential part of care for MSM living with HIV. Second, health care workers and interventionists should focus on MSM with multiple risk behaviors (eg, anal intercourse with NRP, CAI, and multiple male sex partnerships), as these participants were more likely to practice SDU. Those with experience of SDU at baseline also had a much higher risk of practicing SDU during the 6-month follow-up period compared to those who had never practiced SDU. Moreover, MSM who were on PrEP reported higher likelihood of SDU than those not on PrEP. Studies conducted both locally and internationally consistently demonstrated the significant association between SDU and PrEP use [3,24]. This association may be due to the fact that some MSM may continue or initiate SDU as PrEP has minimized their risk of HIV infection. Health care providers supervising MSM on PrEP should continue to provide education about other sexual risks of SDU, such as acquiring STIs, or reduced adherence to PrEP.

Our results showed that MSM in Hong Kong are frequently exposed to information about psychoactive substances and received invitations to use such substances or engage in SDU/chemsex on social media and gay social networking apps. This exposure was associated with a higher prevalence of SDU during the follow-up period. Previous studies consistently showed that social media and gay social networking apps have facilitated access to psychoactive substances, SDU, and chemsex for MSM [18,25,26]. One of the important contributions of this study is that it examined the potential mechanism of the associations between social media and peer influences and SDU. Our results suggest that social media exposure may enhance positive perceptions of SDU, which in turn increase the risk of SDU. The significant mediation effect supports mechanisms proposed by the Social Learning Theory [38] to understand how social media may influence one’s behavior related to SDU. Most social media sites allow users to leave comments, and express approval or disapproval of the contents. Therefore, users can see how many others (and sometimes exactly who) expressed approval when they view social media. This may explain why MSM find social media content to be a believable and influential source of information. Gay social networking apps provide a quick and convenient way to locate and connect with other MSM nearby, and MSM commonly use these apps to seek SDU or chemsex [18,25-28]. Gay social networking app users may perceive SDU as normative as many users may openly solicit SDU or chemsex in their online profiles [18,25,26]. Additionally, receiving a personal invitation of SDU through these apps may make MSM less likely to refuse. Interventions should consider disseminating messages on reducing harm associated with SDU and chemsex on gay apps platforms to reduce perceptions of SDU as normative.

The association between the number of peers in their social network who had experience with SDU/chemsex and SDU was of marginal statistical significance. Previous studies suggested that peers are common sources of obtaining psychoactive substances among MSM [33,34]. It is also common for MSM to introduce and invite peers to have SDU or chemsex [33,34]. However, the TPB perceptions of SDU were not a significant mediator between peer influence and SDU. Other potential theory-based mediators should be explored in future work. We found significant correlations between peer influence and social media influence, and between peer influence and TPB constructs. MSM in Hong Kong contact each other mainly through social media and gay apps. Some of these peers may be online friends that they have never physically met. Since MSM have very close connections to each other, they may find their peers’ experiences particularly valuable [32]. Furthermore, knowing more peers who practice SDU may make MSM perceive such behavior as normative.

These results highlight the importance of modifying perceptions related to SDU based on the TPB. About 20% of the participants held positive attitudes toward SDU. They perceived that SDU would allow them escape from reality, increase their sexual pleasure, and facilitate finding sex partners. Given our findings about the indirect effect of social media influence on SDU behavior, interventions should use social media to shape these attitudes. Health communication messages about reducing substance use during sex and increasing MSM’s awareness of the harms associated with SDU and chemsex can be disseminated using the same keywords used to promote SDU and chemsex, allowing the messages to reach MSM at high risk of SDU and chemsex. Gay social networking apps are also useful for delivering such health communication messages. Some apps widely used in Hong Kong (eg, Blued) have incorporated HIV prevention information and referral to HIV testing services [45]. Although less than 5% of the participants perceived support for SDU from significant others, such perceptions were positively associated with SDU during the follow-up period. Health promotion efforts led by influential peers may be useful to cultivate subjective norms against SDU. Health promotion campaigns may also consider sponsoring support groups led by peers living healthy lifestyles to reduce the influence of substance-using peers. Perceived behavioral control of refraining from SDU needs to be further strengthened as it was a protective factor. Enhancement in self-control skills is warranted and rehearsals may be a useful component of future health promotion programs.

This study had some limitations. First, the results were self-reported and subject to social desirability bias, although anonymity likely reduced the bias compared to nonanonymously collected data. Second, participants were recruited by nonprobabilistic sampling in the absence of a sampling frame. As compared to a representative MSM survey in Hong Kong, our participants had a lower prevalence of HIV and sexual risk behaviors, but higher levels of HIV testing. Third, we were not able to obtain the characteristics of participants who refused to join the study; selection bias might exist. However, the response rate for our study was higher than in other published studies involving MSM in China. Fourth, we did not ask behavioral intention to have SDU or chemsex at baseline. Behavioral intention is an important construct of the TPB that predicts actual behaviors [46]. Furthermore, attrition bias might exist. Those who had lower perceived behavioral control in refraining from SDU were more likely to drop out. Perceived behavioral control in refraining from SDU was a protective factor of SDU. Therefore, the prevalence of SDU during the follow-up period is expected to be higher. Finally, the Negative Attitude Scale had a relatively low Cronbach α (.62) in our sample. Although previous studies suggested that Cronbach α≥.60 was acceptable for exploratory research [47,48], caution is still needed when interpreting the results. Future studies are needed to validate this scale.

In sum, MSM in Hong Kong reported a lower prevalence of SDU and chemsex than that of their counterparts in Western countries. Social media and gay social networking apps may be a major source of influence on MSM’s perceptions and actual behaviors related to SDU, and interventions delivered on these platforms may be especially effective.

## Abbreviations

AOR: adjusted odds ratio
CAI: condomless anal intercourse
CFI: Comparative Fit Index
GHB/GBL: γ-hydroxybutyrate
MSM: men who have sex with men
NNFI: Non-Normed Fit Index
NRP: nonregular partner
PrEP: pre-exposure prophylaxis
RMSEA: root mean square error of approximation
RP: regular partner
SDU: sexualized drug use
STI: sexually transmitted infection
TPB: Theory of Planned Behavior
